# P02.23. The efficacy of prolotherapy using dextrose-morrhuate for lateral epicondylosis: a pilot randomized controlled trial

**DOI:** 10.1186/1472-6882-12-S1-P79

**Published:** 2012-06-12

**Authors:** D Rabago, M Ryan, K Lee, A Chourasia, M Sesto, A Zgierska, D Miller, R Kijowski, J Wilson

**Affiliations:** 1 University of Wisconsin, Department of Family Medicine, Madison, USA

## Purpose

Chronic lateral epicondylosis (CLE) is common, expensive and debilitating. A substantial number of patients are refractory to existing therapy and “watchful waiting.” Prolotherapy is a CAM injection therapy for chronic musculoskeletal pain including tendinopathy. We assessed dextrose prolotherapy for CLE in a pilot-level study.

## Methods

The study design was a 2-arm non-blinded randomized controlled trial. Group 1 received dextrose prolotherapy and Group 2 was a waitlist control. Nineteen adults seen with at least 3 months of symptomatic CLE in 22 elbows refractory to prior care received ultrasound-guided injections of 20% dextrose-morrhuate sodium (Group 1) solution at baseline, 4, and 8 weeks. Waitlist subjects (Group 2) were followed and discouraged from starting new care. Primary outcome measure was Patient-rated Tennis Elbow Evaluation [PRTEE, (100 points) assessed at baseline, 4, 8 and 16 weeks]. Prolotherapy participants were additionally assessed at 32 weeks. Secondary measures included dynamometer-assessed pain free grip strength and participant satisfaction.

## Results

No baseline differences existed between the groups in gender, duration of elbow pain, prior therapy or baseline PRTEE scores. Prolotherapy participants (n=10) reported improved PRTEE composite scores compared to Waitlist (n=12) at 4 and 16 weeks (p<0.05), and improved pain and function PRTEE subscale scores (p<0.05) at 4 and 16 weeks, respectively. Prolotherapy participants reported improvement in composite PRTEE scores from baseline at 16 and 32 weeks of 17.9±11.64 and 24.8±10.58 points, a difference of 49.7% and 70.2% respectively, far in excess of the 11-point PRTEE-based minimal clinical important difference. Grip strength improved in all groups without between-group difference. Satisfaction with prolotherapy was high; there were no adverse events.

## Conclusion

Prolotherapy using dextrose and morrhuate sodium resulted in safe, significant, sustained improvement of PRTEE-based elbow composite, pain and function scores compared to baseline status and waitlist control subjects. The results of this pilot study suggest the need for a definitive clinical trial.

